# Healthcare Costs and Life-years Gained From Treatments Within the Advancing Cryptococcal Meningitis Treatment for Africa (ACTA) Trial on Cryptococcal Meningitis: A Comparison of Antifungal Induction Strategies in Sub-Saharan Africa

**DOI:** 10.1093/cid/ciy971

**Published:** 2019-03-13

**Authors:** Tao Chen, Lawrence Mwenge, Shabir Lakhi, Duncan Chanda, Peter Mwaba, Síle F Molloy, Adrian Gheorghe, Ulla K Griffiths, Robert S Heyderman, Cecilia Kanyama, Charles Kouanfack, Sayoki Mfinanga, Adrienne K Chan, Elvis Temfack, Sokoine Kivuyo, Mina C Hosseinipour, Olivier Lortholary, Angela Loyse, Shabbar Jaffar, Thomas S Harrison, Louis W Niessen, ACTA Trial Team

**Affiliations:** 1 Liverpool School of Tropical Medicine, United Kingdom; 2 Zambart, Health Economics Unit, Lusaka Apex Medical University, Zambia; 3 University Teaching Hospital, Lusaka Apex Medical University, Zambia; 4 Institute for Medical Research and Training, University Teaching Hospital, Lusaka Apex Medical University, Zambia; 5 Department of Internal Medicine and Directorate of Research and Post-Graduate Studies, Lusaka Apex Medical University, Zambia; 6 Centre for Global Health, Institute for Infection and Immunity, St George’s University of London, United Kingdom; 7 London School of Hygiene and Tropical Medicine, United Kingdom; 8 Malawi-Liverpool-Wellcome Trust Clinical Research Programme, Blantyre; 9 College of Medicine, University of Malawi, Blantyre; 10 University College London, United Kingdom; 11 University of North Carolina Project-Malawi, Kamuzu Central Hospital, Lilongwe; 12 Hôpital Central Yaoundé/Site Agence Nationale de Recherche sur le Sida Cameroun, Yaoundé Hopitaux de Paris, France; 13 University of Dschang, Cameroon; 14 National Institute for Medical Research, Muhimbili Medical Research Centre, Dar Es Salaam, United Republic of Tanzania; 15 Dignitas International, Zomba Central Hospital, Malawi; 16 Division of Infectious Diseases, Department of Medicine, Sunnybrook Health Sciences Centre, University of Toronto, Ontario, Canada; 17 Douala General Hospital, Cameroon; 18 Institut Pasteur, Molecular Mycology Unit, Paris, France; 19 University of North Carolina, Chapel Hill; 20 Paris Descartes University, Necker Pasteur Center for Infectious Diseases and Tropical Medicine, Imagine Institute, Assistance Publique – Hopitaux de Paris, France; 21 Department of International Health, Johns Hopkins School of Public Health, Baltimore, Maryland

**Keywords:** cryptococcal meningitis, antifungal induction treatments, cost-effectiveness, flucytosine, sub-Saharan Africa

## Abstract

**Background:**

Mortality from cryptoccocal meningitis remains high. The ACTA trial demonstrated that, compared with 2 weeks of amphotericin B (AmB) plus flucystosine (5FC), 1 week of AmB and 5FC was associated with lower mortality and 2 weeks of oral flucanozole (FLU) plus 5FC was non-inferior. Here, we assess the cost-effectiveness of these different treatment courses.

**Methods:**

Participants were randomized in a ratio of 2:1:1:1:1 to 2 weeks of oral 5FC and FLU, 1 week of AmB and FLU, 1 week of AmB and 5FC, 2 weeks of AmB and FLU, or 2 weeks of AmB and 5FC in Malawi, Zambia, Cameroon, and Tanzania. Data on individual resource use and health outcomes were collected. Cost-effectiveness was measured as incremental costs per life-year saved, and non-parametric bootstrapping was done.

**Results:**

Total costs per patient were US $1442 for 2 weeks of oral FLU and 5FC, $1763 for 1 week of AmB and FLU, $1861 for 1 week of AmB and 5FC, $2125 for 2 weeks of AmB and FLU, and $2285 for 2 weeks of AmB and 5FC. Compared to 2 weeks of AmB and 5FC, 1 week of AmB and 5FC was less costly and more effective and 2 weeks of oral FLU and 5FC was less costly and as effective. The incremental cost-effectiveness ratio for 1 week of AmB and 5FC versus oral FLU and 5FC was US $208 (95% confidence interval $91–1210) per life-year saved.

**Clinical Trials Registration:**

ISRCTN45035509.

**Conclusions:**

Both 1 week of AmB and 5FC and 2 weeks of Oral FLU and 5FC are cost-effective treatments.

## Teaser

Compared to 2 weeks of amphotericin B (AmB) and flucystosine (5FC), both 1 week of AmB/5FC and 2 weeks of oral fluconazole (FLU) and 5FC save costs; 1 week of AmB/5FC reduces mortality rates more and is somewhat more costly.

Mortality from cryptoccocal meningitis (CM) remains high in resource-limited settings [1]. The international standard induction treatment of 2 weeks of amphotericin B deoxycholate (AmB) plus flucytosine (5FC) [2] is not available, while the alternative of fluconazole (FLU) monotherapy is associated with mortality of 50–60% at 10 weeks and >70% at 1 year [3–5].

The ACTA trial [6] tested new induction strategies, based on promising phase 2 data. Both 2 weeks of oral combination therapy with FLU plus flucytosine and 1 week of AmB with either FLU or 5FC were compared against the internationally-recommended 2 weeks of AmB with either FLU or 5FC, in a 2:1:1:1:1 ratio. The aim was to improve upon the efficacy of FLU monotherapy with regimens that, unlike 2 weeks of AmB, could be more readily sustained in resource-limited settings. The trial showed that 1 week of AmB and 5FC was associated with lower mortality and the oral combination was non-inferior compared with the recommended 2 weeks of AmB and 5FC.

Given the scarcity of resources, detailed evidence on the health-care costs of treatment and on the associated health impacts are essential to inform policy decisions. AmB is intravenous and requires hospitalization and stringent laboratory monitoring, while FLU is oral and available through donation programs or as low-cost, generic options. The current availability of 5FC is very limited.

To date, there are very few detailed studies of the costs of alternative CM treatments. Therefore, within the ACTA trial, we conducted a comparative cost-effectiveness study of the 5 regimens tested, in order to support and guide policy decisions.

## METHODS

The ACTA trial [6] was an open-label, phase 3, randomized, non-inferiority, multi-center trial that enrolled patients with human immunodeficiency virus (HIV)-associated CM from 9 African centers in 4 countries (Malawi, Zambia, Tanzania, and Cameroon) between January 2013 and November 2016. Participants were first randomized to 1 of 3 strategies: an oral combination regimen, 1 week of AmB, or the standard 2 weeks of AmB. Those in the AmB arms were further randomized to 5FC or FLU, in a 1:1 ratio, as the partner drug treatment. This resulted in 5 arms, with a ratio of 2:1:1:1:1: (1) 2 weeks of oral 5FC and FLU; (2) 1 week of AmB and FLU; (3) 1 week of AmB and 5FC; (4) 2 weeks of AmB and FLU; and (5) 2 weeks of AmB and 5FC.

A full economic costing and cost-effectiveness analysis of the CM treatments was done, using the consolidated health economic evaluation reporting standards appraisal guidelines [7], from the health-care perspective. Resource use data were collected using an ingredients-based approach [8, 9]. The data on individual resource use and health outcomes, including trial-related complications and treatment of complications, were collected from all participants onto case-report forms. A detailed costing study was done in the Zambian hospital (Table 1 and Supplementary Material). CM-specific and overhead costs, including costs of admissions and laboratory tests, were collated from the hospital’s financial and utilization documents. The treatment-related utilization data were collated from the case-report forms. Data were collected on lengths of stays in hospitals, types of diagnostic tests, and the medical supplies and drugs used. Discussions were held with relevant hospital staff for data triangulation. The ACTA study team were consulted on the trial-related expenditure and resource-utilization data, in relation to complications. Where unit costs were not available in the expenditure records, local market prices were used.

**Table 1.** Unit Prices (US$ in 2015) by Resource Item and Source of Unit Price

Resource Item	Supplies	Staff	Capital	Total	Source
**Costs per bed-day**	9.45	37.06	1.14	47.64	Costing study
**Lumber puncture, per time**	0.94	7.49	1.14	9.57	Costing study
**Biochemistry, per test**					
Total bilirubin	1.9	2.63	0.27	4.8	Costing study
C-reactive protein	5.77	2.63	0.27	8.67	Costing study
Alanine transaminase	4.04	2.63	0.27	6.94	Costing study
Magnesium	2.05	2.63	0.27	4.95	Costing study
Urea	1.94	2.63	0.27	4.84	Costing study
Creatinine	1.83	2.63	0.27	4.73	Costing study
Proteinuria	1.96	2.63	0.27	4.86	Costing study
**Microbiology, per test**					
Urine culture: negative	1.5	5.92	0.43	7.84	Costing study
Urine culture: positive	2.08	8.65	0.43	11.16	Costing study
Blood culture: negative	1.03	6.17	0.55	7.74	Costing study
Blood culture: positive	3.39	9.79	0.55	13.73	Costing study
Sputum culture: negative	2.13	4.4	0.45	6.98	Costing study
Sputum culture: positive	4.04	8.02	0.45	12.5	Costing study
CSF: negative	9.06	13.15	0.46	22.66	Costing study
CSF: positive	9.98	15.88	0.46	26.31	Costing study
**Full blood count, per test**	32.28	4.04	0.28	36.59	Costing study
**CD4 count, per test**	7.38	9.04	1.37	17.79	Costing study
**CM-specific treatment**					
**Trial drug**					
Fluconazole per 1200 mg	0.55			0.55	Provider
Flucytosine per 500 mg	1.53			1.53	Provider
Amphotericin B per 1 mg	1.18			1.18	Provider
**Antibiotics**					
Flucloxacillin per day	0.2			0.2	Pharmacy
Gentamicin per day	0.26			0.26	Pharmacy
Ceftriaxone per ampoule	0.52			0.52	Pharmacy
Amoxicillin/ampicillin per ampoule	0.066			0.066	Pharmacy
Doxycycline per day	0.038			0.038	Pharmacy
Erythromycin per day	0.144			0.144	Pharmacy
Ciprofloxacin per day	0.10			0.10	Pharmacy
**Other intervention**					
Potassium	0.105			0.105	Pharmacy
Magnesium	1.45			1.45	Pharmacy
**Blood transfusion per unit**	35			35	Hospital department
Potassium	2.90			2.90	Costing study
Sodium	2.90			2.90	Costing study

Abbreviations: CM, cryptoccocal meningitis; CSF, cerebrospinal fluid.

A time-and-motion study was conducted to inform the monetary valuation for care provided by health staff at the bedside. It collected information on the types and intensities of care received by a purposive sample of 59 trial participants. Each participant was observed for 2 consecutive days. Findings on the time spent on patient care were combined with salaries (including all financial benefits) to estimate the total staff costs spent on caring for CM patients (Table 1). We collected data on health-care resource and unit prices, adjusted them to the 2015 US$ price level, and included the effects of bulk purchasing and delivery/shipping charges. An average annual exchange rate for the trial baseline year and subsequent inflation corrections were used in the currency conversion and inflation correction.

Aggregated hospital expenditures were allocated proportionally to relevant institutional units and departments. This was complemented with observations to establish cost-allocation factors (eg, floor surface, number of beds, number of medical staff), in particular in the allocation of overhead costs. These additional costs were disaggregated and summarized in costs per bed-day; CM treatment–specific costs and laboratory test costs, according to recurrent and capital costs; and non-specific costs of additional use of antibiotics in relation to complications. Recurrent cost items were considered to be goods/services with a life span of less than 1 year, whereas capital costs were defined as costs which were incurred to purchase good/services that last for more than 1 year. Capital costs were few, were limited to those items related to diagnostics, and were annualized over their economic life (informed by the Zambian hospital’s accounting documents), using a discount rate of 3% [9, 10]. The analysis included institutional and department overheads, including for hospital administration, drugs, and other supply chain management. A detailed listing is given in Table 1, while a more detailed description of the cost component is presented in the Supplementary Material.

The health outcome included in the cost-effectiveness analysis is life-year saved, based on the ages of the patients saved from dying. Here, we multiplied the additional deaths prevented with the observed, CD4-specific, weighted life expectancies [11]. The average life expectancy of the additional survivors was estimated conservatively at 18 years [11]. We did not make a long-term quality-of-life adjustment, as the mortality reduction was substantial and was defined as the main outcome of the trial. Quality-of-life outcomes after CM meningitis in these patient groups are lacking. We used a differential discount rate for health-care costs (3% per international standard) and life-years gained (0%, as given in the literature) [12, 13].

### Statistical Analysis

Total cost—that is, observed use of resources, multiplied by a specific unit price, and increased for specific overhead costs—was adjusted using a Kaplan–Meier average estimator to account for censoring from death or loss to follow-up [14]. Individual patient costs were calculated and non-parametric bootstrapping was used to draw a stable sample (defined as the percentage change in standard deviation between 2 subsequent samples [15]) from patient records by treatment arm to allow for the skewed distribution of costs and the correlation between costs and effectiveness [16].

The 95% confidence intervals for the total cost per patient and the probability of death were calculated using the bias-corrected percentile acceleration method [15]. The 5 ACTA treatments were ranked by their increasing costs, and we ruled out strategies that were less effective and more costly than the comparator (less economically attractive or dominated in economic terms) and strategies that were less effective and had a higher incremental cost-effectiveness ratio (extended dominance). Of the remaining strategies, incremental cost-effectiveness ratios were calculated for each strategy, relative to the standard treatment and the next best alternative.

Costs per life-year saved were estimated by dividing mean incremental costs by mean number of life-years saved. Cost-effectiveness planes and an acceptability curve were used to show the uncertainties around incremental cost-effectiveness ratios. A series of (1-way) sensitivity analyses were done, varying 1 parameter at a time to address uncertainty in the data inputs (including, especially, the observed uncertainty range around patient-level resource use shown in Table 2 and the uncertainties in the observed mortality rate and observed range of life expectancy: 12.8 to 40.81) [17] to compute the uncertainties in incremental cost-effectiveness ratios [18]. Here, the parameters in the standard treatment arm were kept constant. The effects of the top-ranking individual parameters are presented by a tornado sensitivity graph.

**Table 2.** Mean (SD) Resource Use per Patient by Trial Arm, Over 10-Week Trial Period

Service Use Item	Service Use Item	2 Weeks of Oral FLU and 5FC	1 Week of AmB and FLU	1 Week of AmB and 5FC	2 Weeks of AmB and FLU	2 Weeks of AmB and 5FC
**Hospitalization**	Days	17.33 (15.29)	17.14 (18.04)	17.99 (15.06)	16.09 (12.27)	19.31 (18.31)
**Re-hospitalization**	Days	2.02 (5.23)	2.14 (6.31)	0.88 (2.72)	1.77 (5.48)	1.38 (4.10)
**CM-specific treatment**						
***Trial drug***						
Fluconazole	Tablet (200 mg)	187.96 (123.83)	147.18 (130.65)	161.50 (148.25)	170.68 (125.26)	120.37 (159.31)
Flucytosine	Tablet (500 mg)	131 (56)	0.00 (0.00)	74 (23)	0.00 (0.00)	131 (59)
Amphotericin B	Vial (50 mg)	0.00 (0.00)	6.50 (2.60)	7.35 (2.12)	12.71 (5.53)	13.07 (5.94)
** *Antibiotics***						
Flucloxacillin	Times	0.03 (0.17)	0.06 (0.24)	0.05 (0.23)	0.13 (0.34)	0.09 (0.28)
Gentamicin	Times	0.02 (0.13)	0.00 (0.00)	0.01 (0.09)	0.02 (0.13)	0.03 (0.18)
Ceftriaxone	Ampoule	0.62 (0.49)	0.65 (0.48)	0.58 (0.50)	0.60 (0.49)	0.66 (0.48)
Amoxicillin/ampicillin	Ampoule	0.06 (0.24)	0.06 (0.24)	0.11 (0.31)	0.07 (0.26)	0.04 (0.20)
Doxycycline	Times	0.01 (0.09)	0.05 (0.21)	0.00 (0.00)	0.04 (0.18)	0.01 (0.09)
Erythromycin	Times	0.00 (0.00)	0.01 (0.09)	0.01 (0.09)	0.01 (0.09)	0.00 (0.00)
Ciprofloxacin	Times	0.05 (0.22)	0.03 (0.16)	0.04 (0.21)	0.04 (0.21)	0.01 (0.09)
** *Other intervention***						
Potassium	Days	0.00 (0.00)	6.05 (2.20)	6.72 (1.43)	11.71 (4.41)	11.83 (4.62)
Magnesium	Days	0.00 (0.00)	6.05 (2.20)	6.72 (1.43)	11.71 (4.41)	11.83 (4.62)
**Blood transfusion**	Units	0.12 (0.52)	0.23 (0.66)	0.15 (0.57)	0.31 (0.73)	0.37 (0.86)
**Lumbar puncture**	Times	3.13 (1.84)	2.62 (1.07)	3.26 (1.39)	2.93 (1.59)	2.98 (1.44)
**Bio-chemistry**						
Total Bilirubin	Times	1.69 (2.95)	1.57 (2.77)	1.74 (2.99)	1.44 (2.79)	1.63 (2.94)
CRP	Times	0.06 (0.31)	0.09 (0.39)	0.04 (0.21)	0.11 (0.42)	0.10 (0.41)
ALT	Times	3.48 (2.09)	3.04 (1.73)	3.69 (2.00)	3.66 (2.32)	3.40 (1.99)
Magnesium	Times	0.19 (0.73)	0.20 (0.75)	0.20 (0.67)	0.16 (0.66)	0.22 (0.81)
Potassium	Times	7.00 (3.15)	6.23 (3.21)	7.22 (2.35)	7.07 (3.12)	6.83 (3.21)
Sodium	Times	7.02 (3.09)	6.25 (3.21)	7.27 (2.41)	7.12 (3.14)	6.90 (3.23)
Urea	Times	6.99 (3.12)	6.22 (3.14)	7.19 (2.44)	7.04 (3.07)	6.79 (3.12)
Creatinine	Times	7.15 (3.17)	6.32 (3.32)	7.39 (2.45)	7.18 (3.12)	6.98 (3.29)
Proteinuria	Times	7.64 (3.48)	6.82 (3.80)	7.82 (2.71)	7.87 (3.63)	7.47 (3.65)
**Full blood count**	Times	4.62 (2.39)	4.26 (2.62)	4.68 (1.96)	4.69 (2.41)	4.63 (2.60)
**CD4 count**	Times	0.97 (0.37)	1.01 (0.37)	0.93 (0.29)	0.97 (0.45)	0.98 (0.30)
**Microbiology** ^**a**^						
Urine culture: negative	Times	0.08 (0.34)	0.07 (0.32)	0.04 (0.19)	0.11 (0.36)	0.09 (0.45)
Urine culture: positive	Times	0.05 (0.26)	0.04 (0.23)	0.02 (0.13)	0.04 (0.18)	0.02 (0.13)
Blood culture: negative	Times	0.12 (0.36)	0.08 (0.27)	0.10 (0.33)	0.12 (0.44)	0.10 (0.40)
Blood culture: positive	Times	0.07 (0.27)	0.09 (0.35)	0.04 (0.19)	0.13 (0.45)	0.04 (0.24)
Sputum culture: negative	Times	0.00 (0.00)	0.00 (0.00)	0.01 (0.09)	0.00 (0.00)	0.00 (0.00)
Sputum culture: positive	Times	0.07 (0.27)	0.09 (0.35)	0.07 (0.29)	0.06 (0.28)	0.08 (0.27)
CSF: negative	Times	0.64 (0.88)	0.77 (0.99)	1.27 (1.04)	0.75 (0.84)	1.34 (1.21)
CSF: positive	Times	2.44 (1.88)	1.83 (1.14)	1.95 (1.39)	2.17 (1.63)	1.56 (1.02)

Abbreviations: 5FC, flucytosine; ALT, alanine aminotransferase; AmB, amphotericin B; CM, cryptoccocal meningitis; CRP, C-reactive protein; CSF, cerebrospinal fluid; FLU, fluconazole; SD, standard deviation.

^a^Negative cultures are less costly than positive cultures.

### Ethics

The trial protocol and data collection were approved by the London School of Hygiene and Tropical Medicine Research Ethics Committee and by the national ethics and regulatory bodies in each country. Written informed consent was obtained from all participants or, in the case of those with altered mental statuses, from the next of kin (the participants were re-consented on recovery).

## RESULTS

The ACTA trial analysis comprised 678 eligible participants [6]. Only 4 patients were lost to follow-up. The total mortality at 10 weeks (Table 3) was 251 (37%) overall, and was lowest for 1 week of AmB and 5FC (24%, 95% confidence interval [CI] 16–31) [6].

**Table 3.** Probabilistic Cost-effectiveness Analyses, Comparing the Trial Arms in Terms of Mean Total Health Care Costs and Death Rate (%)

	Total Cost per Patient and Death Rate (%) Per Arm		Incremental Comparison of 1 Week of AmB and 5FC Versus 2 Weeks of FLU and 5FC		
ACTA Treatment Arms	Mean Total Costs	Deaths (%)	Incremental Costs per Patient	Incremental Death Rate (%)	Incremental Costs Per Life-year Saved
2 weeks of oral FLU and 5FC	1442 (1336–1565)	35 (28–41)	Reference	Reference	Reference
1 week of AmB and FLU	1763 (1567–1979)	49 (39–58)	...	...	...
1 week of AmB and 5FC	1861 (1724–2033)	24 (16–31)	419 (236–619)	11 (0.6–21)	208 (91–1210)
2 weeks of AmB and FLU	2125 (1946–2313)	41 (32–49)	...	...	...
2 weeks of AmB and 5FC (Comparator)	2285 (2070–2525)	38 (29–46)	...	...	...

Compared to other treatment combinations, 2 weeks of oral treatment and 1 week of AmB and 5FC showed lower costs and better health outcomes (ie, cost less and averted more deaths). In economic terms, these 2 treatments dominate the other options. The incremental cost-effectiveness is shown for these remaining favorable options on the right half of the table. The average estimated life expectancy is 18 years, as reported in Rajasingham et al’s study [11]. The numbers in parentheses are estimates of the 95% confidence intervals, as estimated by boot-strapping. Abbreviations: 5FC, flucytosine; AmB, amphotericin B; FLU, fluconazole.

### Resource Use, Costs, and Health Outcomes

The unit prices are shown in Table 1. The cost per bed-day was $48 in 2015 US$. This excludes CM treatment–specific costs and laboratory test costs. Detailed resource use by trial arm is presented in Table 2. The differences between the trial arms in resource use were largely driven by the component drugs and complication-related resource use. Thus, blood transfusions and potassium and magnesium supplementation were highest for participants in the 2-week AmB arms and lowest for the oral 5FC and FLU combination. The duration of hospitalization was largely similar between the trial arms, as this was protocol-driven. Participants were asked to remain in the hospital as inpatients for at least 14 days for trial safety monitoring.

Mean per patient total costs were lowest for the oral 5FC and FLU combination (US $1442) and highest for 2 weeks of AmB and 5FC (US $2285; Table 3). The total cost of bed-days per patient (both from hospitalization and re-hospitalization) was the major cost component, ranging from 36% of the costs for 2 weeks of AmB and FLU to 56% of the costs for the oral arm. More than 75% of the costs were incurred during the first 2 weeks.

### Cost-Effectiveness and Uncertainty

**Figure 1.**
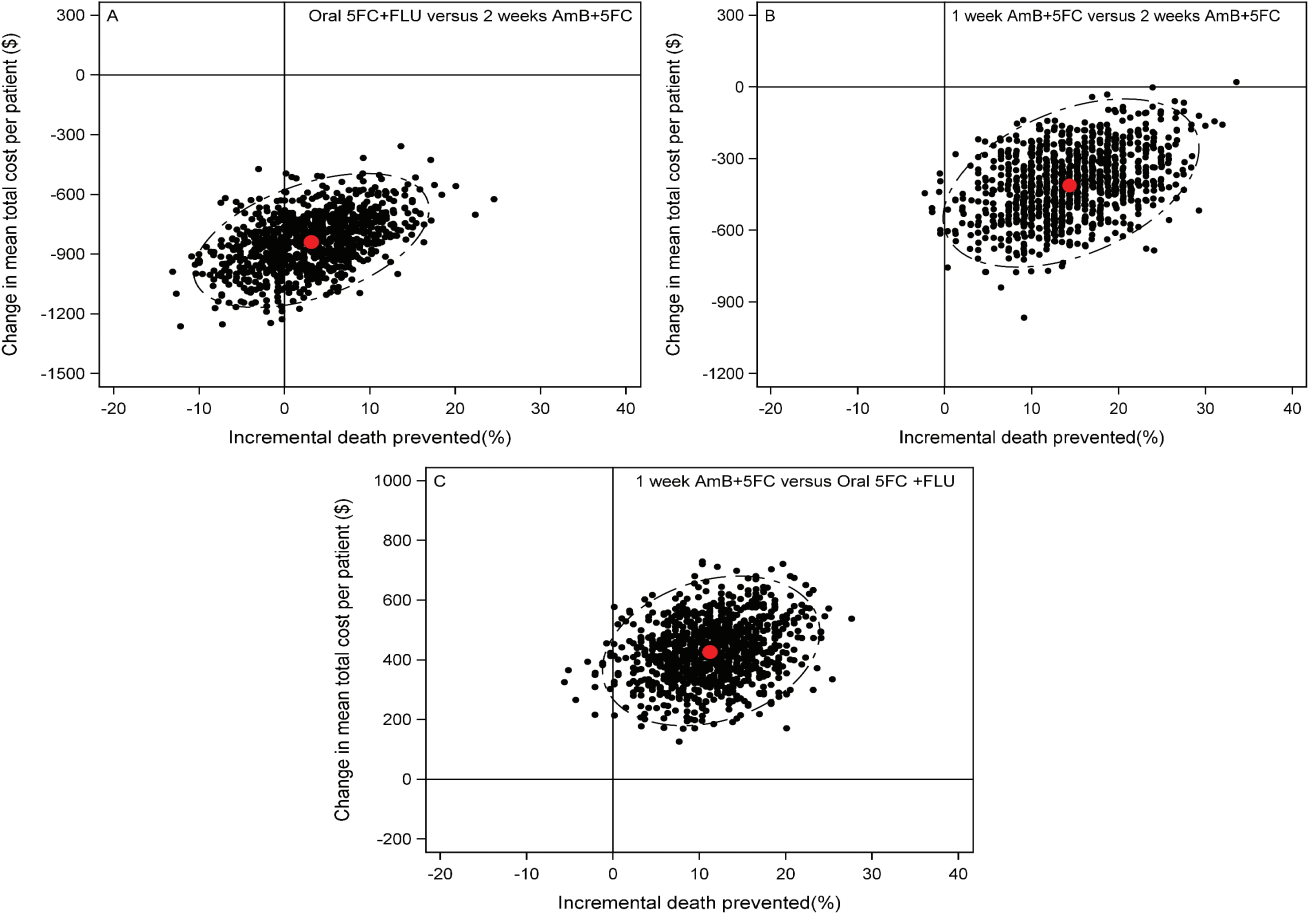
Cost-effectiveness planes after bootstrap iterations (1000 selected at random are shown) to present incremental costs and death prevented (%) after the 10-week trial period, for (*A*) oral 5FC and FLU versus 2 weeks of AmB and 5FC, (*B*) 1 week of AmB and 5FC versus 2 weeks of AmB and 5FC, and (*C*) 1 week of AmB and 5FC versus oral FLU and 5FC. The ellipses show 95% confidence intervals. The red dots indicate the means for both axes. Abbreviations: 5FC, flucytosine; AmB, amphotericin B; FLU, fluconazole.

We determined that 1 week of AmB and 5FC was less costly and more effective than (ie, dominated) 2 weeks of AmB and 5FC (Table 3 and Figure 1). While 2 weeks of oral 5FC and FLU was also less costly than 2 weeks of AmB and 5FC, the reduction in mortality was marginal (Table 3 and Figure 1). Both 2 weeks of AmB and FLU and 1 week of AmB and FLU were cost-saving when compared with 2 weeks of AmB and 5FC, but these treatments were associated with increased mortality (Table 3 and Figure 1).

**Figure 2.**
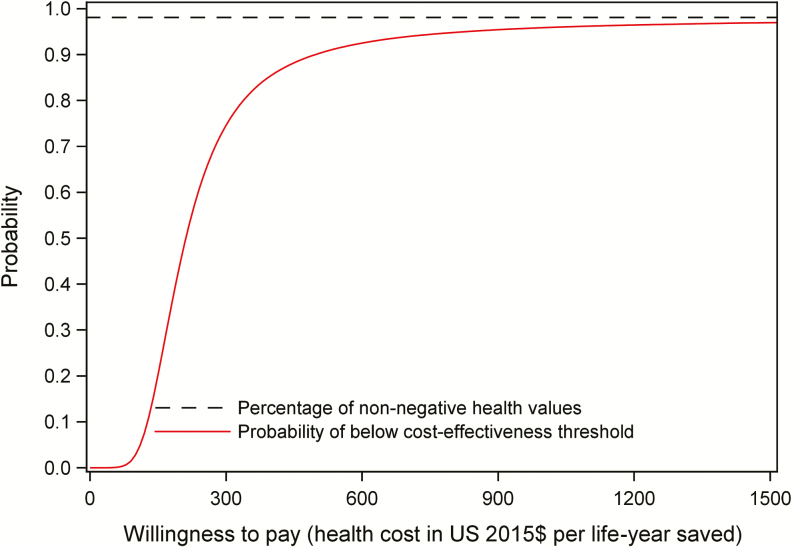
Cumulative probability of the incremental cost-effectiveness ratio being below different thresholds for the comparison of 1 week of AmB and 5FC versus oral FLU and 5FC. Abbreviations: 5FC, flucytosine; AmB, amphotericin B; FLU, fluconazole.

Therefore, 1 week of AmB and 5FC and 2 weeks of oral combination were the 2 most attractive induction treatments. Figure 2 shows the uncertainty around the health-service cost savings, in relation to the number of lives saved, in scatter plots of the cost-effectiveness plane. In comparison to 2 weeks of AmB and 5FC, 2 weeks of oral 5FC and FLU is robustly cost saving, but the health gain is much less certain. In comparison, 1 week of AmB and 5FC shows a robust reduction in both cost and deaths. Finally, in a head-to-head comparision (Table 3, right side) 1 week of AmB and 5FC shows a robust health gain, at some additional cost, compared with the oral 5FC and FLU regimen (US $208 per life-year gained, 95% CI $91–1210).

Figure 2 shows the probability (y-axis) that 1 week of AmB and 5FC is cost-effective when compared with 2 weeks of oral 5FC and FLU at the complete, full range of willingness-to-pay thresholds (x-axis). The probability of a course of treatment being cost-effective exceeds 90% at a threshold of US $490 and is around 80% at a threshold of US $330 per life-year saved.

### Multi-variate Sensitivity Analysis

**Figure 3.**
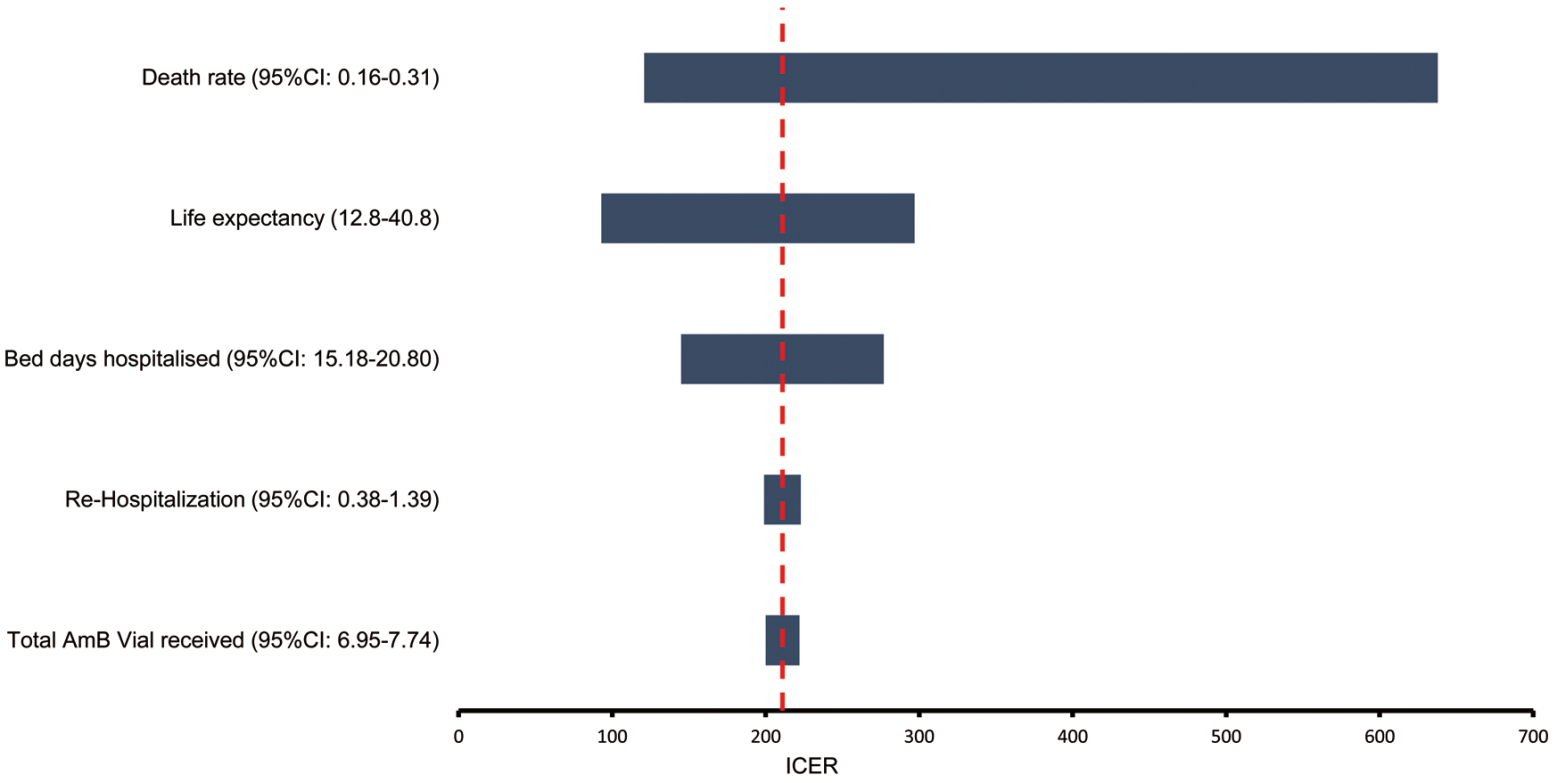
Tornado diagram of ICER for 1 week of AmB and 5FC vs an oral combination for major components. All other resource parameters were not influential. The analyses use the 95% of the input distribution for resource use and health outcomes parameters to eliminate extreme outliers. The input ranges are based on the overall results from bootstrap methods described in the methods section, using individual participant data. Life expectancy input data are from a comparable cohort [11, 17] Abbreviations: 5FC, flucytosine; AmB, amphotericin B; CI, confidence interval; ICER, incremental cost-effectiveness ratio.

We varied all the resource parameters (Table 2) and the health outcomes (Table 3) in an empirical, multi-variate sensitivity analysis (Figure 3). The top 5 drivers of the incremental cost-effectiveness ratio were mortality rate, life expectancy, number of bed-days hospitalized, number of re-hospitalization days, and total AmB dosage. The latter and all other parameters did not substantially influence the incremental cost-effectiveness results. The tornado graph in Figure 3 shows the effect of varying the value for each important parameter on the incremental cost-effectiveness ratio of 1 week of AmB and 5FC, compared with the oral regimen, given the uncertainty ranges in the individual parameters in probabilistic analyses. If the mortality for 1 week of AmB and 5FC was varied from 16% to 31% (ie, the lower and upper 95% CI of the mortality estimate), then the incremental cost-effectiveness ratio would vary between $121 and $638, assuming other parameters were constant.

## DISCUSSION

This study shows that 1 week of AmB and 5FC and oral FLU and 5FC were the most cost-effective regimens, and either is suitable to replace 2 weeks of AmB and 5FC as the preferred regimen in many settings. In comparison with 2 weeks of AmB and 5FC, both regimens were less costly and 1 week of AmB and 5FC led to substantial health gains, while the oral combination was at least as effective.

The findings for 1 week of AmB and 5FC were very robust. Even when the mortality of 1 week of AmB and 5FC was varied to the upper 95% CI limit of 31%, 1 week of AmB and 5FC still dominated 2 weeks of AmB and 5FC. In an arm-to-arm comparison, the estimated incremental cost-effectiveness ratio for 1 week of AmB and 5FC versus oral 5FC and FLU was $208 per life-year saved. In clinical settings in Africa where AmB can be given and monitored, 1 week of AmB and 5FC represents a cost-effective option. Importantly, however, oral fluconazole and flucytosine provides a cost-effective option for more severely resource-limited settings, where AmB therapy is not possible; that is, it is as effective as the current international standard of care and reduces service costs. The findings re-emphasize the absolute necessity of current international efforts to secure immediate and wide access to flucytosine.

This is the first large study based on the collection of protocol-driven, patient-level resource-use data across different African countries. These data were supplemented with medical and nursing staff time information and an empirical costing study in the Zambian public hospital site. Here, as in many other sub-Saharan African countries, costs data are not included in routine health-service data collection. Therefore, as part of ACTA, we undertook substantial efforts to obtain reliable, consistent, and accurate data on components of the service costs per bed-day, the main driver of total costs per patient. The resulting cost per bed-day was relatively high (US $48): it reflected the real-life local cost of intensive treatment and local procurement, and excluded CM treatment–specific costs and specific laboratory-test costs.

In a prior study to compare the cost-effectiveness of alternative regimens, Rajasingham et al concluded that 1 week of AMB plus fluconazole would likely be much more cost-effective than 2 weeks of AmB courses [11]. A limitation acknowledged by the authors was that the efficacy component was based on the pooled mortality data available at that time across small, often non-comparative studies and from different settings. Our service costs and resource-use data are linked to the largest trial to date. The results confirm the economic attractiveness of 1 week of AmB in relation to 2 weeks of AmB, in terms of cost savings and higher effectiveness, but only in combination with 5FC as the partner drug. They also demonstrate the attractiveness of the combination of the 2 oral drugs: fluconazole and 5FC. The 1-week course with AmB comes at an additional price compared wth oral FLU and 5FC, which may be affordable in many sub-Saharan Africa country settings, and compares well with a range of other clinical interventions, including the prevention of mother-to-child HIV transmission, multidrug-resistant tuberculosis treatment, and intrapartum care [18–20].

The cost-effectiveness advantage of 1 week of AmB and 5FC and oral FLU and 5FC, as compared to 2 weeks of AmB regimens, is underestimated in our analysis. We measured actual durations of hospitalization of the ACTA trial participants, which, for trial safety monitoring reasons, required participants to be hospitalized under close observation for the first 2 weeks. In real-life implementation, the duration of hospitalization for patients on either regimen would, in all likelihood, be lower; therefore, the cost of these regimens would decrease in relation to 2 weeks of AmB regimens, which require a minimum duration of hospitalization of 14 days. If we included all societal cost consequences, including those at the household level, the total societal cost savings of either regimen over 2 weeks of AmB regimen would increase further, as travel and loss of household productivity in relation to hospitalization would be reduced, as well as the out-of pocket patient-related costs born by carers.

In an explorative scenario, we subtracted the cost of the second week’s admission from the total for any patient discharged on day 14 or earlier who was on oral treatment or on 1 week of AmB and 5FC (using the original trial data). This shorter hospital-stay scenario results in a total per patient cost of US $767 (95% CI 722–841) for the oral arm and US $1161 (95% CI 1114–1225) for 1 week of AmB. These costs are about half those of the per-protocol hospital stays for patients on these arms and are substantially lower than the costs of 2 weeks of AmB and 5FC ($2285), making both regimins even more attractive. Importantly, the incremental cost-effectiveness of 1 week of AmB and 5FC versus the oral combination would not be altered by this consideration, since all CM patients require some period of hospitalization for optimal care, including the measurement and management of raised cerebrospinal fluid pressure.

This study provides further strong support for the recently-updated World Health Organization guidelines for the treatment of HIV-associated CM, which recommend 1 week of AmB and 5FC and oral FLU and 5FC as the first and second preferred regimens. Flucytosine needs to be made available widely to reduce cryptococcal-associated mortality.

## Supplementary Data

Supplementary materials are available at *Clinical Infectious Diseases* online. Consisting of data provided by the authors to benefit the reader, the posted materials are not copyedited and are the sole responsibility of the authors, so questions or comments should be addressed to the corresponding author.

ciy971_suppl_Supplementary_Material

## Notes

***Acknowledgments*.** The authors thank all the patients and their families, as well as the staff at all the sites not directly involved in the trial. They thank Andrew Nunn, Halima Dawood, Andrew Kitua, and William Powderly for serving on the data and safety monitoring committee; Graeme Meintjes, Calice Talom, Newton Kumwenda, and Maryline Bonnet for serving on the trial steering committee; and the Agence Nationale de Recherche sur le Sida (ANRS) Staff in Paris (Brigitte Bazin, Claire Rekacewicz, and Paula Garcia) for constant support.

***Authors’ contributions***. T. C. analyzed the data. T. C. and L. M. wrote the first draft of the manuscript. L. W. N. supervised the analysis. T. S. H., S. J., and L. W. N. led the writing and interpretation of data; A. G. and U. K. G. provided input. T. S. H. led the design of the study; S. L., D. C., P. M., M. C. H., O. L., and S. J. provided input. L. M. led the costing study in Zambia; S. L., D. C., and P. M. provided input. S. F. M. provided oversight and monitored the data collection. C. Kanyama, C. Kouanfack, E. T., S. K., R. S. H., and A. L. were responsible for the data collection and study supervision. All authors contributed to drafts of the paper and approved the final version.

***Financial support.*** This work was supported by grants from the Medical Research Council in the United Kingdom (grant number 100504), the French Agency for Research on AIDS and Viral Hepatitis (Agence Nationale de Recherche sur le Sida grant number ANRS12275), and a strategic award from the Wellcome Trust UK (to the Malawi–Liverpool–Wellcome Clinical Research Programme).

***Potential conflicts of interest.*** T. S. H. reports grants from Medical Research Council and Agence Nationale de Recherche sur le Sida, during the conduct of the study; grants and personal fees from Gilead Sciences; personal fees from Pfizer and Viamet; and non-financial support from Immunomycologics, outside the submitted work. O. L. reports personal fees from Pfizer, Gilead, Astellas, and Merck, outside the submitted work. All other authors declare no potential conflicts. All authors have submitted the ICMJE Form for Disclosure of Potential Conflicts of Interest. Conflicts that the editors consider relevant to the content of the manuscript have been disclosed.
