# Supplementary material for: Healthcare Costs and Life-years Gained From Treatments Within the Advancing Cryptococcal Meningitis Treatment for Africa (ACTA) Trial on Cryptococcal Meningitis: A Comparison of Antifungal Induction Strategies in Sub-Saharan Africa
Source: Clin Infect Dis. 2019 Mar 13;69(4):588–95. doi: 10.1093/cid/ciy971 (PMC6669289; doi:10.1093/cid/ciy971)
Supplement: ciy971_suppl_Supplementary_Material [file ciy971_suppl_supplementary_material.docx]

Online Supplemental Material for the following article

**Annex to Health care costs and deaths prevented by ACTA trial treatments for cryptococcal meningitis: a comparison between five induction strategies in sub Saharan Africa**

**ACTA Trial Team**

In addition to the authors listed in the paper, the following were members of the ACTA trial team:

*Malawi-Liverpool-Wellcome Trust/University of Malawi/Queen Elizabeth hospital, Blantye, Malawi* – W. Chimanganga, E. Chilima, E. Dziwani, E. Gondwe, P. Goodson, C. Jassi, C. Kukacha, L. Keyla, M. Likwinj, D. Ming, D. Segula, A. Singimi, S. Miyango, V. Mwloza, L. Matandika, N. Mthunthama, J. Ndaferankhande, C. Rothe, C. Sambakunsi, F. Shumba, N. Kalata, K. Gaskell, M. Peirse. J. Ellis,

*General Hospital, Douala, Cameroon –* E. Bagna, F. Bella, A. Dang, J. Epupa, H. Essaka, G. Goula, H. Luma, A. Ngoula, Y.N. Mapoure.

*UNC Project, Kamuzu Central Hospital, Lilongwe, Malawi* – T. Banda, B. Banda, T. Chikaonda, M. Chikasema, M. Chipeta, N. Chome, C. Kanyemba, E. Kasonkanji, E. Kumwenda, W. Kumwenda, W. Mhango, L. Msumba, P. Mwangomba, F. Namaluweso, J. Phulusa, I. Shumba, D. Sichali, C. Chawinga, J.J. van Oosterhout, C. van der Horst.

*Institute for Medical Research and Training, University Teaching Hospital, Zambia* – D. Chanda, A. Chansa, E. Chikatula, Y. Kakusa, M. Mputu, M. Mukasine, J. Mwaba, W. Mwambazi, L. Mwanamoonga, A. Mweeba, J. Nglube, M. Sichone, J. Silumbe, A. Tande, N. Karunaharan

*National Institute Medical Research, Muhimbili Medical Research Centre, Dar es Salaam, Tanzania -* A. Abdallah, D. Buma, A.A. Chande, E. Diarz, M. Hassan, J.M. Kalabashanga, R. Kalinga, D. Kileo, A. Kimambo, R. Lawrence, T. Massawa, M. Mganga, S. Mamboya, M.M. Nyange, K. Okeng’o, R. Simbaulanga, J. Rugemalila, I.B. Tarimo.

*Hȏpital Central Yaoundé/Site ANRS Cameroun* - S. Kouala-Shiro, E. Delaporte, M. Alma, R. Dombu, R.E. Djoukwe, C. Embolo, S. Le Gac, A. Kamdem, G. Mounpou, E.P. Owono, E. Pasquier, E. Sonkeng, S. Tongo, P. Vigne, M. Wodo, V. Sini, S. Lontsi, J-G. Ndong

*Dignitas International, Zomba Central Hospital, Zomba, Malawi* - G. Kaphale, E. Nyondo, A. Jusu, K. Kalima, Y. Mataka, C. Khoriyo, L. Silwamba, P. Bright, D. Lupiya.

*Centre for Global Health, Institute for Infection and Immunity, St George’s University of London, London, United Kingdom* - S. Abdelmalik, S. Burton, N. Stone, J. Adams, T.Bicanic.

*London School of Hygiene and Tropical Medicine, London, United Kingdom* - V. Simms, J. Bradley

*Liverpool School of Tropical Medicine, Liverpool, UK*. D. Lalloo, D. Wang

***Detailed costing study***

The ACTA economic study is based on the health care perspective, using an ingredient approach. This means that the total cost of the resource used in the delivery of the treatment is based on measured detailed use of medical resources per individual patient in each of trial arms and this is multiplied by the unit price in the local setting in US$2015.

*Per bed-day cost computations*

Per bed-day cost were calculated by combining medical wards overhead costs and ward-specific costs, and then divide by the total in-patient days. Overhead costs were calculated in one trial sites, the hospital in Zambia. Medical ward overheads were estimated by dividing total hospital overheads by established allocation ratios. To calculate allocation ratios, we divided appropriate medical wards and laboratory values by total hospital values – for equipment, building and furniture we divided medical ward surface by total hospital surface area. For transportation equipment costs allocation, we divided total number of staff working in medical wards by total number of staff at the hospital. For recurrent cost allocation, utilities and equipment maintenance costs were apportioned by dividing medical ward surface by total hospital surface area while personnel and transport overhead costs were allocated by dividing total number of staff in medical wards by total number of hospital staff. Then the ratios were multiplied by total hospital expenditures to calculate total hospital overhead allocation for medical wards.

An ingredient costing approach was used to calculate ward-specific costs. Both medical and non-medical supplies were quantified and valued. Resource quantification was done by reviewing purchase and requisition documentations. Resource valuation was done by combining these quantities and unit costs established from hospital and Medical Stores Limited price lists. Annual quantities were calculated through review of purchase and supply records which was done in close collaboration with staff in the procurement department. Total annual costs for medical wards was computed by combining unit cost/price and annual quantity information to calculate total ward-specific costs. To calculate ward specific personnel costs, we summed up annual ward specific costs from personnel and salary report for the hospital in 2012, adjusted for the year 2015.

Cost data was collected on expenditures, length of stay in hospital, types of diagnostic tests, medical supplies, drugs and service utilization data through table review in collaboration with hospital administration and finance department. Data sources were financial records, procurement documents and patient records.

# *Treatment cost computations*

Like for per patient day cost calculation, an ingredient costing approach was used to calculate treatment costs. Specific drugs to treat cryptococcal meningitis (CM), supplementary drugs (antibiotics and other complication related drugs) and related supplies were quantified from CM patient clinical records in collaboration with ACTA trial team. Valuation was done by combining these quantities and unit costs established from ACTA trial expenditure documentations. Cost data was collected on trial expenditures, length of stay in hospital, types of diagnostic tests, resource utilization, treatment regimen and supplementary treatment.

# *Laboratory tests*

Cost analysis was also performed for cryptococcus meningitis-related laboratory tests. Per test costs were calculated by combining laboratory overhead costs and test-specific costs. Overhead and shared costs were divided by the total throughput at each procedure. Laboratory costs were also divided into laboratory recurrent, personnel, overhead, and capital costs. Average overhead costs were calculated by dividing the annual “overhead” costs of running the laboratory department by the annual number of tests performed to deduce an approximate estimate of overhead costs per test. Capital costs per test were calculated by dividing annualised capital costs by the annual number of tests performed. Recurrent costs per test were calculated by combining utilization data and units. Finally, overhead costs per test, capital costs per test and recurrent costs per test were summed up to arrive at average total costs per test.

Like for Medical ward overheads calculation, we divided total hospital overheads by established allocation ratios to estimate the laboratory overhead costs. Allocation ratios were calculated by dividing laboratory values by total hospital values. For Equipment, building and furniture we divided Laboratory surface area by total hospital surface area. For transportation equipment allocation total number of laboratory staff by total number of hospital staff. For recurrent cost allocation, utilities and equipment maintenance costs were apportioned by dividing Laboratory surface area by total hospital surface area while personnel and transport overhead costs were allocated by dividing total number of staff in the laboratory by total number of staff at hospital. Then the ratios were multiplied by total hospital expenditures to calculate total overhead costs allocated to the laboratory department.

Laboratory (lab) tests-specific costs were calculated using an ingredient costing approach through which cost items for each procedural step were identified, quantified and valued. Cost items were identified through observation, review of SOPs and interviews with laboratory staff. Resource quantification was done by count the number of units used for each resource - consumables and capital equipment were counted in their physical units whereas staff time was timed to get full time-equivalent value for each procedure. Resource valuation was done by combining the quantities and unit costs established through reviewing purchase and requisition documentations at the hospital for 2012. Cost per sample/test at each procedural step was calculated by multiplying quantities per sample with respective unit costs/prices. Per test-specific personnel costs were calculated by multiplying daily salaries with staff time spent (in minutes) for each procedural step and then divided by the employable minutes in a day. For Capital cost per sample/test at each step was calculated by dividing annualised value annual throughput for each test.

*Hospital Overhead*

Detailed overhead costs were based on findings in the one study site, the hospital in Zambia, involved in the study. We used its annual financial status report for 2012. Cost data, including total cost and unit cost were computed based on expenditures, total hospital throughput, medical ward throughputs, laboratory throughput and space data through table review in close collaboration with hospital administration and finance department. Top-down analysis was used to allocate overhead costs. Equipment, building and furniture were allocated by surface area. Transportation equipment were allocated by number of staff. Utilities and maintenance costs were apportioned by surface area while personnel and recurrent transport costs were allocated by number of staff. The hospital is the largest hospital in Zambia with in-patient capacity of 2,000 bed across 56 wards. The hospital has four main clinical departments; medicine, obstetrics and gynaecology, paediatrics and surgery. Furthermore, the hospital has the following supportive departments: radiology, laboratories, nursing services, occupational health, pharmacy and physiotherapy. The department of internal medicine has 6 wards with an occupancy rate of 71%. The department admits around 84,571 patients per year representing about 12% of total admission.

*Cross-country validation*

The ACTA clinical trial and the trial treatments are strictly protocolised we expected resource use across countries not to be very different, given the selected treatment and its potentially expected (absence of ) complications and standardized treatment of complications. We have used the collected resource data across the five trial arms for each country to pool the resource data across country to strengthen the internal validity of the resource computations. The resulting differences in health service cost by week and by trial arm do not show big differences between the Zambia results and the results for the other countries combined.
